# PI3K-Akt signaling pathway based on network pharmacology for the anti-Alzheimer’s disease effect of licorice stem flavonoids

**DOI:** 10.18632/aging.204536

**Published:** 2023-02-22

**Authors:** Hongyan Pei, Lei He, Meiling Shi, Xiangjuan Guo, Weijia Chen, Jianming Li, Zhongmei He, Rui Du

**Affiliations:** 1 College of Chinese Medicinal Materials, Jilin Agricultural University, Changchun 130118, Jilin, China; 2 Jilin Provincial Engineering Research Center for Efficient Breeding and Product Development of Sika Deer, Jilin Agricultural University, Changchun 130118, Jilin, China; 3 Key Laboratory of Animal Production and Product Quality and Safety, Ministry of Education, Jilin Agricultural University, Changchun 130118, Jilin, China

**Keywords:** network pharmacology, Alzheimer’s disease, licorice stem flavonoids, PI3K-Akt signalling pathway

## Abstract

Active ingredients were screened by TCMSP and swissADME, meanwhile, PharmMapper combined with UniProt database was used to predict the active ingredient target information, GeneCard database was employed to obtain Alzheimer's disease (AD)-related genes, Cytoscapes 3.7.2 software was utilized to map the active ingredient-target effect. Besides, Cytoscapes 3.7.2 software Bisogenet and Cyto NCA plug-in combined with STRING platform were utilized to map the protein-protein interaction (PPI) network, DAVID was employed for GO annotation, while KEGG plug-in was used for KEGG pathway enrichment. Mice were tested for inflammatory damage induced by intracerebral injection of lipopolysaccharide (LPS), as well as learning memory and anxiety by water maze and open field tests. In addition, the expression of Caspase-3 and Caspase-9, together with inflammatory factors TNF-α, IL-6, and IL-1β was analyzed in serum. The expression levels of proteins related to PI3K-Akt signaling pathway in the brain were detected by Western blot (WB) assay. According to the results of network pharmacology, there were 35 active ingredients of licorice stem and leaf flavonoids screened, which exerted the anti-Alzheimer's disease (AD) effects via 67 targets and activated 41 signaling pathways including the PI3K-Akt pathway. Furthermore, Behavioural results revealed that Licorice stem and leaf flavonoids improved the learning and memory abilities of model mice and significantly improved the anxiety caused by inflammatory brain damage. Moreover, as suggested by HE staining and TUNEL staining of brain sections, Glycyrrhiza glabra stem and leaf flavonoids alleviated morphological lesions and cell nuclear damage in brain tissue. Results: of brain homogenate supernatant assay demonstrated that Glycyrrhiza glabra stem and leaf flavonoids had a significant effect on the levels of oxidative indicators superoxide dismutase (SOD), catalase (CAT), malonaldehyde (MDA), acetylcholine (Ach), acetylcholinesterase (AchE), Caspase-3, Caspase-9 and serum inflammatory factors TNF-α, IL-6 and IL-1β. Additionally, WB assay results indicated that the PI3K-Akt signaling pathway was activated.

## INTRODUCTION

Licorice is the dried root and rhizome of Glycyrrhiza uralensis Fisch, Glycyrrhiza inflata Bat, or Glycyrrhiza glabra L., a leguminous plant that has a wide and long history of clinical use [1, 2]. Licorice stems and leaves are used as the by-products of licorice, mostly in animal husbandry [3, 4]. Notably, licorice stems and leaves are complex in composition and rich in flavonoids [1, 5], and the contained flavonoids possess a variety of pharmacological activities [6].

Network pharmacology can analyze the mechanism of action of ingredients in diseases through network patterns, and serve as a purposeful guide for the mechanistic study of complex ingredients. When analyzing the therapeutic effects of Glycyrrhiza glabra stem and leaf flavonoids on Alzheimer’s disease (AD), a network pharmacology-based approach can clarify the molecular targets and mechanisms of action of Glycyrrhiza glabra stem and leaf flavonoids against AD. The intracerebral injection of lipopolysaccharide (LPS) in mice can lead to neuroinflammation and anxiety-like behavior, while inflammation can in turn trigger acetylcholine abnormalities and oxidative stress, satisfying the cholinergic hypothesis, the inflammatory response theory and oxidative damage as a model of AD, while its anxiety-like behavior can also be used as an indicator of model improvement [7–9]. In this study, the anti-AD effects of licorice stem flavonoids were investigated through behavioral experiments in mice, staining of brain tissue sections, inflammatory factors, oxidative factors, acetylcholine and other indicators.

## RESULTS

### Ingredient acquisition and screening

The results are shown in Table 1. As observed, no active ingredient was found from TCMSP, but swissADME screened the active ingredients with GI absorption = high and Druglikeness: “Yes” ≥2, as displayed in Table 2.

**Table 1:** Active components of TCMSP.

**Molecule ID**	**TCMSP,OB≥30%, DL≥0.18**	**CAS**	**OB (%)**	**DL**
MOL004856	gancaonin A	27762-99-8	51.08	0.4
MOL004857	gancaonin B	124596-86-7	48.79	0.45
MOL005000	gancaonin G	126716-34-5	60.44	0.39
MOL005001	gancaonin H	126716-35-6	50.1	0.78
MOL004863	gancaonin L	129145-50-2	66.37	0.41
MOL004864	gancaonin M	129145-51-3	30.49	0.41
MOL004866	gancaonin O	129145-53-5	44.15	0.41
MOL004961	quercetin-3,3′-dimethylether	4382-17-6	46.45	0.33
MOL002563	galangin	548-83-4	45.55	0.21
MOL004828	glepidotin A	42193-83-9	44.72	0.35
MOL003673	wighteone	51225-30-0	42.8	0.36
MOL003656	lupiwighteone	104691-86-3	51.64	0.37
MOL003398	pratensein	2284-31-3	39.06	0.28
MOL000392	formononetin	485-72-3	69.67	0.21
MOL004910	glabranin	41983-91-9	52.9	0.31
MOL004910	pinocembrin	68745-38-0	46.08	0.18
MOL004328	naringenin	480-41-1	59.29	0.21
MOL005190	eriodictyol	552-58-9	71.79	0.24
MOL004829	glepidotin B	87440-56-0	64.46	0.34
MOL002311	glycyrol	23013-84-5	90.78	0.67
MOL004948	isoglycyrol	23013-86-7	44.7	0.84
MOL000098	quercetin	117-39-5	46.43	0.28

**Table 2:** Active ingredients of swissADME.

**GI absorption =high, Druglikeness:”Yes”≥2**	**CAS**
gancaonin F	
gancaonin p-3′-methylether	
uralenin	139163-17-0
8-prenyleriodictyol	
6-prenyleriodictyol	
5′-prenyleriodictyol	
6-prenglnaringenin	68236-13-5
8-prenglnaringenin	53846-50-7
exiguaflavanone K	
kanzonol S	
sigmoidin C	101923-93-7
pinobanksin	548-82-3
citflavanone	

### Prediction of active ingredient targets

Thirty-seven flavonoid active ingredients from the above-ground parts of Glycyrrhiza glabra were predicted as targets in PharmMapper, then their names were converted and de-duplicated in the Uniprot database to obtain 67 active ingredients as targets.

### AD-related gene target acquisition and screening

**Figure 1:**
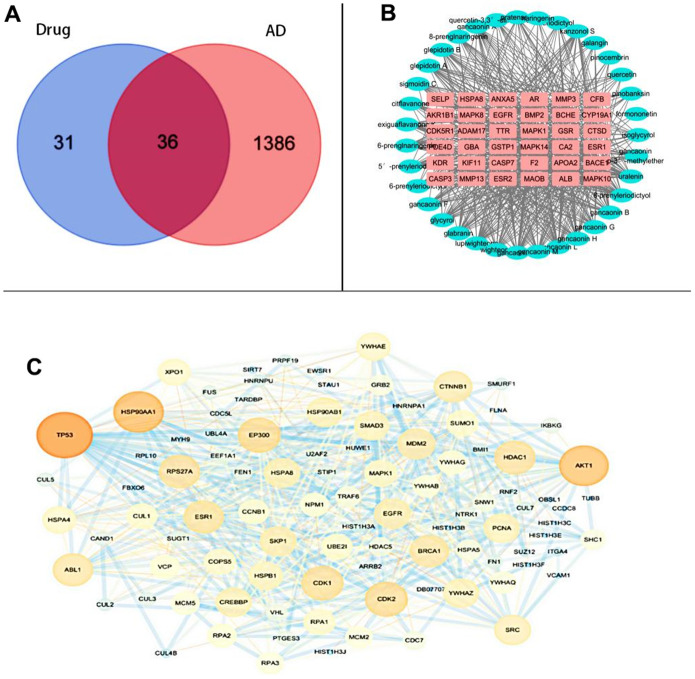
Common-target network (**A**) Intersection targets (**B**) Composition-core target network diagram (**C**) PPI network diagram.

The GeneCard database was searched to yield 10975 AD-related targets, among which, 1422 AD-related targets were screened and de-weighted with a Relevance score ≥10. These targets were later compared with those of active ingredients, and 36 potential targets were acquired from the flavonoids of Glycyrrhiza glabra against AD. The Venn diagram of the intersecting targets is presented in Figure 1A.

### Active ingredient-core target network construction

The 35 active ingredients and 36 intersecting targets were imported into Cytoscapes 3.7.2 software, and thereafter the active ingredient-core target network was plotted, including 71 nodes and 583 edges. As observed from Figure 1B, each component contained multiple targets, and different components might correspond to the same target.

### PPI network construction

Subsequently, 67 active ingredient targets and 1422 screened AD-related targets were imported into Cytoscapes 3.7.2 software Bisogenet plug-in to obtain a PPI network graph. Moreover, the NCA plug-in was utilized to analyze and filter the resulting intersection. The minimum interaction threshold was set at >0.7, and the TSV file was imported into Cytoscapes 3.7.2 software to construct a PPI network regarding the intersection of the PPI network for active ingredient targets of the licorice ground part and the PPI network for AD targets. There were 97 nodes and 604 edges in the graph. The node size and the edge thickness were set by the Degree value and the combined score value, with the larger Degree value indicating the larger node and the larger combined score value representing the thicker edge. The PPI network is displayed in Figure 1C.

### Gene functional annotation and pathway enrichment analysis

**Figure 2:**
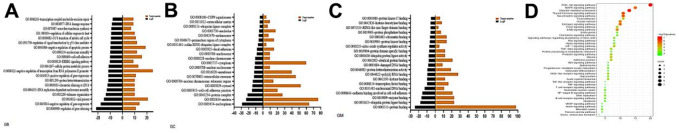
**Enrichment of gene ontology (GO) and KEGG pathway.** (**A**) GB analysis (**B**) GC analysis (**C**) GM analysis (**D**) Bubble diagram of signaling pathway.

DAVID was adopted to perform GO functional annotation and KEGG enrichment analysis on the key nodes obtained from the PPI network. To be specific, the GO functional annotation analysis was divided into biological process (GB), cellular component (GC), and molecular function (GM) categories. According to our results, GB yielded 239 entries, including negative regulation of RNA polymerase II promoter transcription, viral process, positive regulation of RNA polymerase II promoter transcription, negative regulation of apoptotic process, transcription, DNA template, intercellular adhesion, negative regulation of transcription, DNA template, positive regulation of transcription, DNA template, negative regulation of gene expression, and so on. Meanwhile, GC yielded 59 entries, such as protein complexes, intercellular adhesion junctions, nuclear chromosomes, nucleosomes, nucleoli, extracellular matrix, mitochondria, and intracellular ribonucleoprotein complexes. While GM yielded 77 entries, including protein binding, double-stranded RNA binding, enzyme binding, protein phosphatase binding, and estrogen receptor binding. The top 20 entries, ranked according to P-value from the smallest to the largest, were selected for visual analysis (Figure 2A–2C).

As for KEGG pathway enrichment analysis, there were 41 signaling pathways enriched (Figure 2D), including the common AD-related pathways such as PI3K-Akt signaling pathway, MAPK signaling pathway, ErbB signaling pathway, NF-κB signaling pathway, apoptosis, TNF signaling pathway, and estrogen signaling pathway.

### Morris water maze test

**Figure 3:**
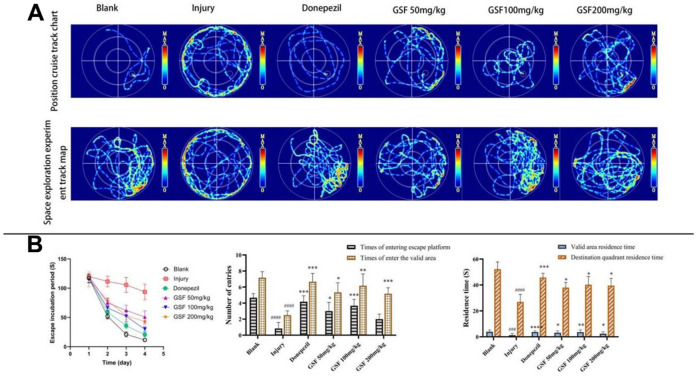
**Morris water maze test.** (**A**) Infrared trace diagram of water maze (**B**) Escape latency, entry times and residence time of mice.

Positioning cruise test: the results are shown in Figure 3. Mice in blank group were able to find the platform quickly, while intracerebral injection of LPS decreased the ability of mice to find the platform, and administration of each dose of licorice stem flavonoid and positive drug increased the ability of mice to find the platform. As observed from Figure 3, the escape latency significantly increased in model group compared with blank group, and significantly decreased in each administration group compared with model group.

Spatial exploration test: the results are presented in Figure 3A. Mice in blank group purposefully searched for the escape platforms in the target quadrant, while those in model group swam purposelessly in each quadrant. Compared with model group, administration of each dose of licorice stem flavonoid and positive drug remarkably increased the number of times that mice entered escape platforms, the number of times they entered the effective area, the effective area residence time and the target quadrant residence time (P < 0.05), as shown in Figure 3B.

### Open field test

**Figure 4:**
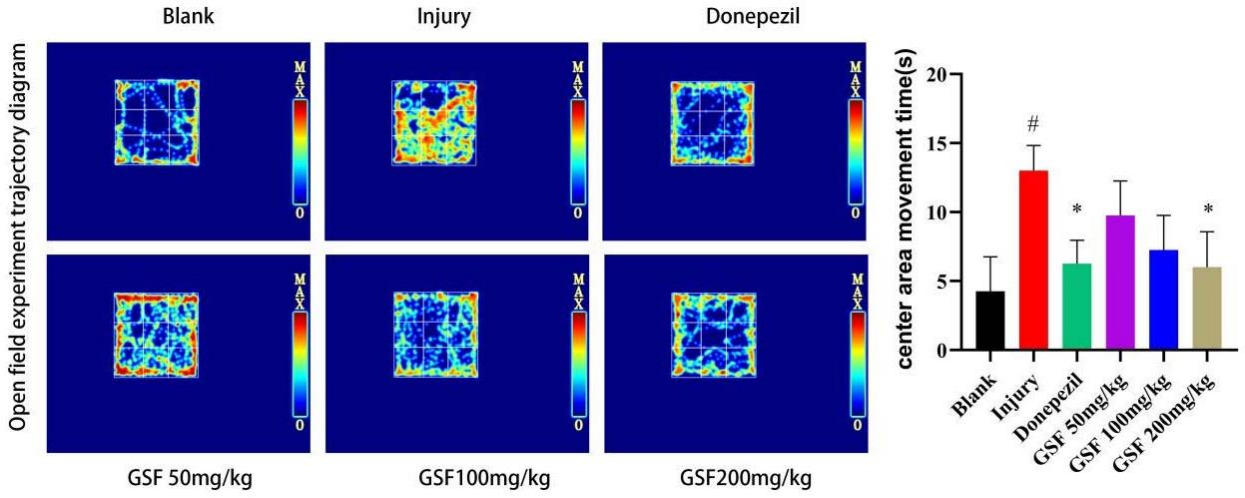
Infrared trajectory diagram of the open field test and movement time statistics in the central area of the open field.

Normal mice tend to move around the edges of the field in an unfamiliar environment due to their avoidance, while anxiety will cause the mice to explore irregularly against their avoidance. The results of open field test are shown in Figure 4. As a result, mice in blank group mainly explored along the edges of the open field, while those in model group showed irregular exploration behavior in the central area. The statistical results indicated that the irregular exploration behavior in the central area was significantly alleviated in each drug administration group and positive drug group compared with model group (P < 0.05).

### Hippocampal staining in mice

**Figure 5:**
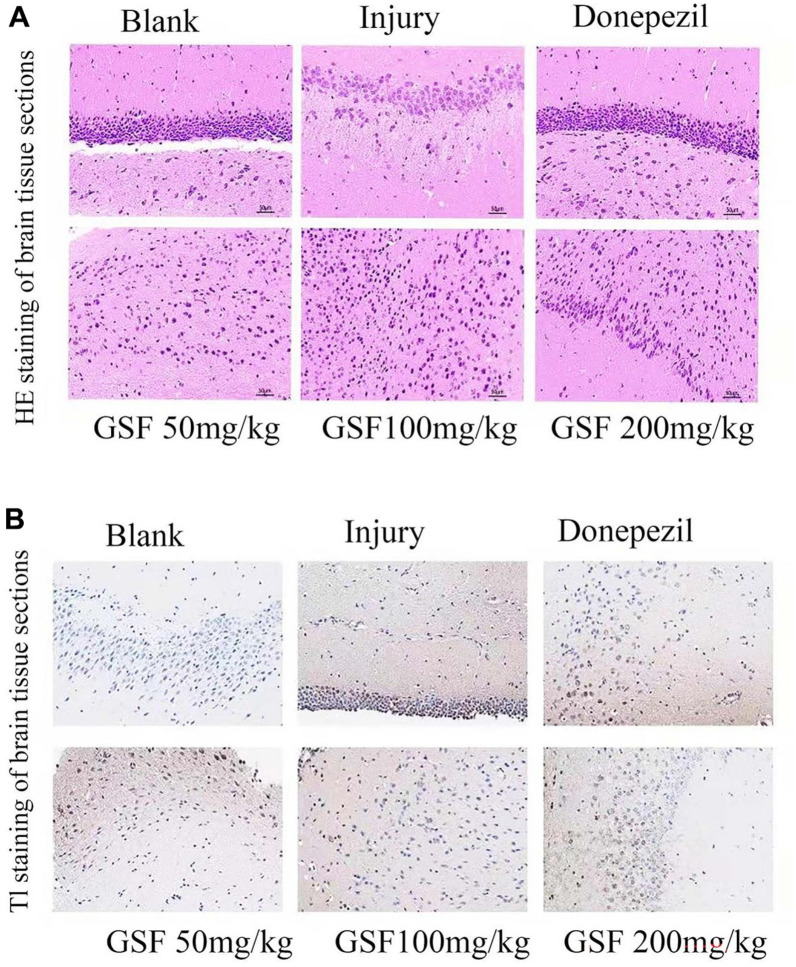
Staining of mouse hippocampal sections (**A**) HE staining of mouse hippocampal sections. (**B**) TUNEL staining of mouse hippocampal sections.

The results of HE staining can be observed from Figure 5A. Clearly, the neuronal cells in the brain of mice in blank group were neatly arranged, with clear nuclei and uniform staining, while those in the brain of mice in model group showed broken cytoplasm, disorganized and loose arrangement, with unclear structure and uneven staining.

The TUNEL staining results are shown in Figure 5B. Obviously, the positive rate of TUNEL staining dramatically increased in model group compared with blank group, and decreased after administration of different concentrations of Glycyrrhiza glabra stem and leaf flavonoids and positive drugs, in other words, Glycyrrhiza glabra stem and leaf flavonoids alleviated the cell nuclear damage caused by LPS.

### Test results of serum TNF-α, IL-6 and IL-1β levels

**Figure 6:**
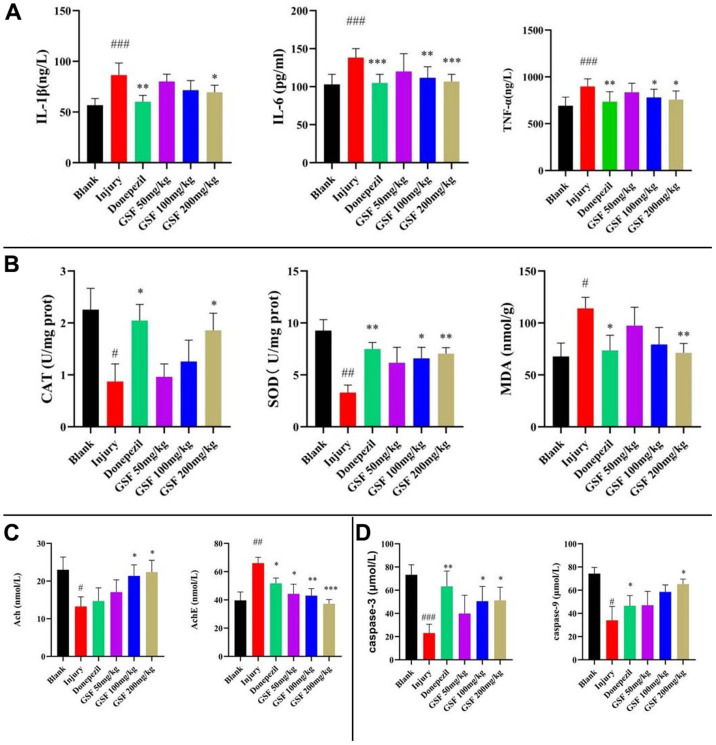
Inflammatory Factor and Oxidative Stress Marker Assays (**A**) Levels of inflammatory factors. (**B**) Brain oxidation indicator level testing. (**C**) Levels of Ach, AchE. (**D**) Contents of Caspase-3 and Caspase-9.

Serum inflammatory factor levels are presented in Figure 6A. Compared with blank group, TNF-α, IL-6 and IL-1β levels in model group dramatically increased (P < 0.05); relative to model group, there was no significant difference in the administration of low dose of licorice stem flavonoids (P > 0.05). Meanwhile, administration of medium dose and high dose of licorice stem flavonoids and positive drug significantly decreased serum TNF-α and IL-6 levels (P < 0.05), and administration of high dose of licorice stem flavonoids and positive drug evidently decreased the serum interleukin levels (P < 0.05).

### Test results of brain oxidation index levels

The brain oxidation indexes are displayed in Figure 6B. Compared with blank group, SOD and CAT activities remarkably decreased in model group (P < 0.05), and MDA content evidently increased (P < 0.05). Compared with model group, administration of high dose of licorice stem and leaf flavonoids and the positive drug markedly reversed SOD and CAT activities (P < 0.05) and evidently decreased MDA content. By contrast, the low dose of licorice stem and leaf flavonoids did not significantly improve the brain oxidation indicators; while the medium dose of licorice stem and leaf flavonoids had a remarkable effect on the recovery of SOD activity, but not on the recovery of CAT activity or the reduction of MDA content (P > 0.05).

### Results of changes in brain AchE and Ach contents

Brain AchE and Ach levels are demonstrated in Figure 6C. Compared with blank group, Ach levels in model group decreased significantly and AchE levels increased evidently (P < 0.05). Relative to model group, the high dose of licorice stem flavonoids group and positive drug group apparently reversed the Ach levels and significantly decreased AchE levels; whereas low and medium doses of licorice stem flavonoids groups did not exhibit significant improvement (P > 0.05).

### Results of brain Caspase-3 and Caspase-9 levels

As shown in Figure 6D, Caspase-3 and Caspase-9 levels significantly decreased compared with blank group (P < 0.05); high dose of Glycyrrhiza glabra stem flavonoids and positive drugs significantly alleviated the reduction in Caspase-3 and Caspase-9 levels relative to model group (P < 0.05).

### PI3K-Akt signaling pathway-related protein expression

**Figure 7:**
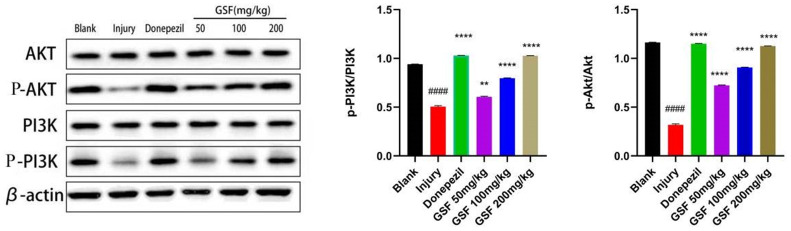
PI3K-Akt signaling pathway protein expression.

Compared with blank group, the p-PI3K/PI3K and p-Akt/Akt ratios in model group decreased markedly (P < 0.0001), and the administration of positive drugs and various concentrations of Glycyrrhiza glabra stem and leaf flavonoids significantly elevated the ratios to varying degrees (P < 0.05). The results are shown in Figure 7.

## DISCUSSION

Network pharmacology can be used to analyze the mechanism of action of ingredients in diseases through network patterns, and it plays a purposeful role in guiding mechanism research of complex ingredients. In this study, 75 major flavonoid components were collected from relevant database, 35 active ingredients were screened and 67 active ingredient targets were identified. Besides, 41 signaling pathways were obtained by constructing the PPI networks and enrichment analysis.

PI3K-Akt signaling pathway is a neuroprotective signaling pathway that is widely present in all types of cells [10], its inhibition and activation are closely related to AD. When the PI3K-Akt signaling is inhibited, the downstream mitochondrial pathway is activated and apoptosis is promoted; by contrast, the activation of PI3K-Akt signaling pathway leads to phosphorylation of the AKT cascade, thereby inhibiting oxidative damage [11–13].

Patients with AD develop progressive memory and cognitive dysfunction, behavioral and personality changes, accompanied by psychiatric symptoms such as apathy, hallucinations and depression. In this study, the effects of licorice stem flavonoids on the pathological behavior of mice with an inflammatory model of AD were investigated by water maze and open field tests. In the water maze test, the model mice lost the spatial memory learning ability that the normal mice had, and licorice stem flavonoids inhibited the loss of memory learning ability in mice. Intracerebral injection of LPS and production of inflammatory factors caused anxiety-like behavior in model mice. In the open field test, model mice were engaged in frequent central area exploration against avoidance, which might reflect their cognitive dysfunction and personality changes [14, 15]. It has been shown that due to the prolonged modeling time, mice may change from anxiety-like to depressive behavior, leading to a decrease in central area exploration behavior in the open field test [16]. In the present study, the mouse anxiety-like behavior in the open field test decreased with increasing concentration of the administered drug.

AD is characterized by cortical atrophy, widening of the sulcal gyrus and loss of neurons [17, 18]. In this study, abnormalities in the structure, arrangement and nuclei of neuronal cells were observed in the brain tissue cells of model mice prepared in brain tissue sections, and TUNEL staining was also conducted to observe the extent of nuclear damage. The licorice stem flavonoids improved the brain pathological features of model mice, which reflected their anti-AD effect.

Oxidative stress is one of the components of the inflammatory response [19]. Studies have shown that oxidative stress plays an important role in the pathogenesis of AD. Oxidative stress and inflammatory response are the critical parts of the pathological process of AD [20, 21]. In this study, by detecting the levels of inflammatory factors and oxidative stress indicators, it was found that licorice stem flavonoids inhibited the inflammatory response. Acetylcholine is an essential neurotransmitter, and its reduced level is one of the important features of AD [22, 23]. It has been discovered that acetylcholine exerts anti-inflammatory effects at the protein level, but not at the transcriptional level [24, 25]. Licorice stem flavonoids can increase acetylcholine content and decrease acetylcholinesterase content in mouse brain, suggesting that they are involved in the release of inflammatory inhibitory factors and the apoptosis of neuronal cells.

Activation of caspases is one of the typical characteristics of apoptosis, but in the late stages of apoptosis and in dead cells, caspases appear to significantly decrease by cleavage activation levels [22, 26]. In this study, the decrease in caspases in the brain of model mice was observed using the caspase-3 and caspase-9 assay kits, besides, the decreasing trend of caspases was weakened in the licorice stem and leaf flavonoids group, suggesting that licorice stem and leaf flavonoids played a protective role against apoptosis.

Moreover, in this study, the effect of licorice stem and leaf flavonoids on the expression of PI3K-Akt signaling pathway-related proteins P-PI3K/PI3K and P-Akt/Akt was examined by WB assay. Consistent with the results of network pharmacological analysis, licorice stem and leaf flavonoids had a therapeutic effect on AD by activating the PI3K-Akt signaling pathway.

## CONCLUSIONS

The results of network pharmacology and animal experiments indicated that Glycyrrhiza glabra stem flavonoids inhibited neuronal apoptosis through the activation of PI3K-Akt signaling pathway, which was achieved possibly by regulating acetylcholine, inhibiting oxidative stress, inflammatory response, and modulating Caspase-3 and Caspase-9, thus improving the learning and memory abilities of mice.

## MATERIALS AND METHODS

### Databases

Databases including Pubchem (https://pubchem.ncbi.nlm.nih.gov/), TCMSP (http://tcmspw.com/tcmsp.php/), swissADME, (http://www.swissadme.ch/), PharmMapper (http://www.lilab-ecust.cn/pharmmapper/), Uniprot (https://www.uniprot.org/), GeneCard (http://www.genecards.org/), STRING (https://cn.string-db.org/), DAVID (https://david.ncifcrf.gov/) were used.

### Animals

A total of 60 female Kunming mice weighing 23-27 g were housed at the room temperature of 20-25° C and the relative humidity of 40-70%, with free access to food and water.

### Drugs and materials

Licorice stem and leaf flavonoids (laboratory-made (purity ≥ 67%), LPS, SOD assay kit, CAT assay kit, MDA assay kit, Caspase3 assay kit, Caspase9 assay kit (Beijing Solabao Technology Co., Ltd.), 0.9% saline (Kunming Nanjiang Pharmaceutical Co., Ltd.), titanium dioxide (Shanghai Maclean Biochemical Technology Co. Ltd.), 10% formaldehyde fixative (laboratory preparation), TNF-α assay kit, IL-6 assay kit, IL1-β assay kit, Ach assay kit, AchE assay kit (Jiangsu Enzyme Immunity Industry Co., Ltd.), electronic balance FA21041 (Shanghai Jingtian Electronic Instruments Co., Ltd.), frozen centrifuge 1-16K (Sigma-Aldrich Shanghai Trading Co., Ltd.), CNC ultrasonic cleaner KH-300DB type (Kunshan Hechuang Ultrasonic Instrument Co., Ltd.), Morris water maze (Chengdu Taimeng Technology Co., Ltd.), Absence field (Chengdu Taimeng Technology Co., Ltd.), Microplate spectrophotometer (BioTek Instruments, USA), and Embedding machine (Leica, Germany) were utilized in this study.

### Methods

### Ingredient acquisition and screening

The SMILES and two-dimensional (2D) chemical structures of the constituents were obtained from Pubchem by searching relevant literature for the above-ground flavonoid constituents of Glycyrrhiza glabra. Thereafter, the active ingredients were screened on the TCMSP platform for oral bioavailability (OB) and drug likeness (DL) values using the ingredient names and CAS numbers, and those not found on the TCMSP platform were screened on swissADME using the 2D structure diagrams.

### Prediction of active ingredient targets

The 2D molecular structures of active ingredients were uploaded to PharmMapper as an SDF file, with the targets restricted to “Human Protein Targets Only”. The UniProt IDs of the obtained targets were then converted into Gene Symbols in the Uniprot database.

### AD-related target acquisition and screening

AD-related targets were searched in the GeneCard database, then the search file was downloaded, filtered and de-duplicated using the Relevance score attribute. Later, the resulting disease targets were compared with the active ingredient targets, and a Venn diagram of the intersecting targets was created.

### Active ingredient-core target network construction

Thereafter, the active ingredients and intersecting targets were imported into Cytoscapes 3.7.2 software to map the active ingredient-core target network.

### Protein-protein interaction (PPI) network construction

The PPI network was constructed by importing the active ingredient targets and the AD-related targets into the Cytoscapes 3.7.2 software Bisogenet plug-in. Later, the PPI intersection network was obtained by taking the intersection of the two plots, and then the PPI intersection network of licorice stem flavonoids and AD was obtained. Four attribute values of Degree, betweenness, Closeness, and LAC were later utilized to perform a second screening. The obtained key nodes were thereafter imported into STRING, and the minimum interaction threshold was limited to > 0.7. Afterwards, the PPI network maps were obtained and imported into Cytoscapes 3.7.2 software as TSV files, so as to construct the PPI maps regarding the intersection of PPI networks for active ingredient targets of the above-ground parts of Glycyrrhiza glabra and the PPI networks for AD targets.

### Gene functional annotation and pathway enrichment

The key nodes obtained from the PPI network were imported into DAVID for Gene Ontology (GO) annotation analysis and Kyoto Encyclopedia of Genes and Genomes (KEGG) enrichment analysis, and the species was restricted to “Homo sapiens” with a threshold of P-value < 0.05. Graph Pad Prism was employed to visualize the analysis.

### Animal modeling and drug delivery

After one week of acclimatization, the mice were randomly divided into six groups, including blank group, model group, low dose group, medium dose group, high dose group and positive drug group.

Mice in each group were injected with 5 g/L LPS solution (3 μL/mouse) in the lateral ventricle using a stereotaxic instrument (Nanjing Kevin Biotechnology Co., Ltd.), while those in control group were given injection of saline. Meanwhile, licorice stem and leaf flavonoids were administered by gavage at 200 mg/kg, 100 mg/kg and 50 mg/kg in the high, medium and low groups, respectively. Donepezil hydrochloride was administered at 3 mg/kg by gavage in the positive group, and saline was administered by gavage in the blank and model groups. The drug was administered for 21 days in total.

### Morris water maze test

On day 14 of administration, the Morris water maze was used to assess the spatial memory and learning ability of mice. To be specific, the water maze system consisted of a cylindrical pool (diameter, 120 cm; height, 60 cm), an escape platform, a camera apparatus and a system for analyzing the behavioral trajectories of mice, which was surrounded by shade. The water maze was divided into four quadrants, and mice were randomly selected to be placed in one of these four quadrants, and the time for each mouse to find the platform within 120 s was considered as the escape latency period. If the platform was not found within the specified time, the mice were guided to stay on the platform for 30 s. Subsequently, the mice were trained for 4 days and different quadrants were selected each day. At the end of the training period, mice were placed in any quadrant for a positioning cruise experiment, and the time required to find the platform within the time limit of 60 s was recorded. Thereafter, the escape platform was removed and a spatial exploration test was performed. The number of times that the mice entered the escape platform, the number of times that they entered the effective area, the time they stayed in the effective area and the time they stayed in the target quadrant within 120 s were recorded, respectively.

### Open field test

The open field test set-up consisted of a black isolation box (dimension, 30 x 30 cm) and a camera system. Notably, the open field apparatus was cleaned before each experiment to avoid odor affecting the mice. The mice were placed into the box at a fixed point, then their trajectory and the time spent in the central area were monitored for 3 min.

### Sample collection and processing

At the end of the behavioral experiments, blood was collected from eyes, placed in the refrigerator overnight at 4° C, and centrifuged at 4° C and 4000 r/min to extract the serum. Meanwhile, brain tissues were taken, ground on ice with 200 μL RIPA lysate per 100 mg, and centrifuged to extract the supernatant. Thereafter, the two brain tissues from each group were placed in 10% formaldehyde fixative and set aside.

### Staining of brain tissue sections

Brain tissues were fixed in 10% formaldehyde fixative, dehydrated in gradient ethanol solution (70%, 80%, 90%, 95% and 100% in succession), then transparentized in xylene, paraffin-embedded and sectioned for analysis. Subsequently, HE staining was performed on the paraffin sections in line with specific instructions to observe the histopathological changes in brain tissue of each group. Moreover, TUNEL staining was performed on the paraffin sections according to specific instructions to detect the apoptosis of brain tissue in each group.

### Inflammatory factor and oxidative stress marker assays

The ELISA kits were designed to detect the levels of TNF-α, IL-6 and IL-1β; SOD, CAT and MDA; acetylcholine and acetylcholinesterase; caspase-3 and caspase-9 in the supernatants of mouse brain tissue homogenates.

### Western blot (WB) assay for the expression of proteins related to the PI3K-Akt signaling pathway in mouse brain

The BCA Protein Concentration Kit was designed to detect the protein concentrations in the supernatants of brain tissue homogenates. To be specific, one sample with a final concentration of 1 mg/mL of protein was prepared using PBS and 5X sampling buffer, which was mixed well and boiled in boiling water for 3-5 min to denature the proteins in chains. After separation by polypropylene amide gel electrophoresis (PAGE), the samples were transferred to polyvinylidene difluoride (PVDF) membranes, and the excess was cut off according to the marker and protein molecular weight. Later, the membranes were incubated with 5% skimmed milk powder, primary antibodies and secondary antibodies in turn.

### Statistical analysis

All data were expressed as mean ± SEM and analyzed by one-way or two-way ANOVA with Tukey’s test for comparison between multiple groups. A p-value < 0.05 was considered significant.
